# Mechanical performance of steel reinforcing bars in uncorroded and corroded conditions

**DOI:** 10.1016/j.dib.2018.04.072

**Published:** 2018-04-25

**Authors:** Silvia Caprili, Walter Salvatore

**Affiliations:** University of Pisa, Department of Civil and Industrial Engineering, Italy

## Abstract

The paper presents data coming from a wide experimental test campaign executed on different typologies of steel reinforcing bars representative of the actual European production scenario. Tensile and low-cycle fatigue tests have been executed to assess the mechanical performance of reinforcing bars under monotonic and cyclic/seismic conditions. The effects of exposure to aggressive environmental conditions have been reproduced through accelerated salt-spray chamber. Residual mechanical performance of corroded specimens has been analyzed as function of corrosion indicators such as mass loss and necking.

**Specifications table**

**Table:** 

Subject area	*Engineering*
More specific subject area	*Earthquake engineering*
Type of data	*Tables, figures*
How data was acquired	*For monotonic and low-cycle fatigue (LCF) tests: forces were acquired from load cell, deformations through displacement sensors. Measurement of mass loss and of necking on corroded specimens were acquired manually.*
Data format	*Processed and analyzed*
Experimental factors	*Steel reinforcing bars were produced and preliminary tested to assess their conformity to standards before being sent to laboratories.*
Experimental features	*Monotonic stress–strain curves from tensile tests; cyclic stress–strain curves from LCF tests in corroded and uncorroded conditions. Micrography of reinforcing steels through SEM analysis.*
Data source location	*Italy, Europe.*
Data accessibility	*Data is with the article.*
Related research article	*Caprili S. & Salvatore W. Cyclic behaviour of uncorroded and corroded steel reinforcing bars, Construction and Building Materials 76 (2015) 168–186.*

**Value of the data**

• Data provide information about mechanical properties (yielding and ultimate tensile strength, elongation to maximum load, ultimate elongation, hardening ratio, necking) and dissipative capacity (dissipated energy and number of cycles up to failure) of a wide set of steel reinforcing bars. Data can be used to compare differences related to production process, steel grade, ductility class, producer and plant.
• Data provide indications about the effects of corrosion on different typologies of steel reinforcements. Effects are evaluated in terms of decrease of mechanical properties (ductility and strength, dissipative capacity) in relation to the corrosion indicator mass loss.
• Data of corrosion tests can be used to estimate the reduction of performance of existing RC constructions
• Corrosion effects (mass loss, necking) can be compared to data coming from other corrosion procedures representing different exposure conditions.

## 1 Data

Actual European standards for reinforced concrete (RC) constructions [1] prescribe minimum mechanical requirements for reinforcing steels in different delivery conditions (i.e. bars, wires, coils and lattice girders). Differences among production processes, diameters and metallurgical properties are not mentioned. The large variability of standards' requirements leads to about 200 different steel grades able to satisfy Eurocodes' prescriptions for civil constructions.

A set of representative steel grades was selected and tested under monotonic and cyclic loads in uncorroded and corroded conditions providing a global overview of European reinforcing steels’ behavior under static and seismic loading conditions before and after the deterioration due to aggressive environmental conditions. In particular:

• Monotonic tensile tests were executed following EN 15630-1:2010 [1].
• Cyclic tests (i.e. Low-Cycle Fatigue - LCF) adopted a specific protocol elaborated to represent the ductility demand required by the earthquake [3], [4].
• Corrosion effects were reproduced through accelerated tests in salt-spray chamber for different exposure periods, following the procedure presented in [5].

The set of steel reinforcing bars (rebars) includes: different steel grades (B400, B450, B500) and ductility classes (A, B, C according to Eurocode 2 [1]), different diameters (*ϕ*8, *ϕ*12, *ϕ*16, *ϕ*20 and *ϕ*25 mm) and different production processes (TempCore - TEMP, Micro-Alloyed MA, Stretched - STR and Cold-Worked - CW). The variability due to steel makers and plants was considered: specimens were provided by two different European producers, presented in the following as “Prod. 1” and “Prod. 2”; different plants were used (Table 1).

**Table 1:** Representative set of steel reinforcements selected for mechanical characterization.

Steel grade	Ductility	Diameter *ϕ* (mm)	Process	Producer and plant
B500	A	8	CW	Prod. 1
B500	B	16	TEMP	Prod. 1 (3 different plants)
B500	B	8	STR	Prod. 1
B450	C	16	TEMP	Prod. 1 (3 different plants)
B450	C	8	STR	Prod. 1
B400	C	8, 20, 16	TEMP	Prod. 1
B500	A	8, 12	CW	Prod. 2
B500	B	8, 16, 20, 25	TEMP	Prod. 2 (same cast for all diameters)
B450	C	16, 20, 25	TEMP	Prod. 2 (same cast for all diameters)
B450	C	8, 12	STR	Prod. 2
B400	C	16, 20, 25	MA	Prod. 2 (same cast for all diameters)

## 2 Experimental design, materials and methods

### 2.1 Experimental characterization of uncorroded steel reinforcing bars

#### 2.1.1 Metallurgical investigations

**Fig. 1:**
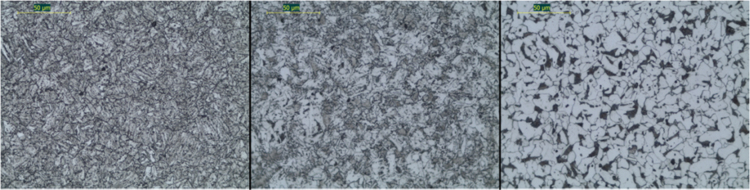
Typical microstructures present in a cross-section of B450C-16-TEMP-2.1 TempCore® reinforcing bar, skin: tempered martensite, intermediate zone: bainite/ferrite mixture, and core: ferrite–pearlite.

**Fig. 2:**
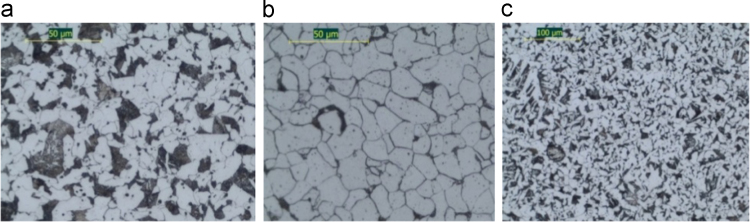
(a) Typical micro structural in a cross-section of B400C-16 (Prod. 2), (b) cross-section of B500A-8-CW (Prod. 2), (c) cross-section of B500B-8-STR (Prod. 1).

Macrographic and metallographic analyses and hardness tests were executed on rebars presented in Table 1. Specimens were prepared for metallographic examinations and etched with 3% Nital solution to determine the hardness profile of bars’ cross-sections. In the case of TempCore® the typical macrostructure consisting of three main concentric zones (a skin of tempered martensite on the surface, an intermediate zone with a mixture of bainite and ferrite and a ferrite–pearlite core) was revealed (Fig. 1). The extensions of skin, intermediate zone and core were evaluated by considering the area of the phases on the metallographic samples (Table 2). Fig. 2 shows the typical microstructure of Micro-Alloyed steels, consisting of pearlite and ferrite; Table 3 summarizes the summary of the microstructural features (Ferrite Grain Size - FGS) and the measured hardness are reported for tested MA, CW and STR specimens. FGS was measured using the intercept method. Each specimen has been provided by a specific tag, used in the following, indicating:

• The steel grade (B400, B450 or B500) and the diameter (in mm).
• The ductility class (A, B or C).
• The production process (TEMP, MA, CW or STR), the producer and the plant.
• The typology of rib (ribbed – R; indented – I).

**Table 2:** Measured bars properties for TempCore® steel reinforcing bars.

Specimen (tag)		Core hardness	Skin hard.	Skin	Intermediate zone	Core diameter
1	B400C-8-TEMP-R-Prod. 1	164.5 HV	257 HV	0.52 mm	0.28 mm	6.79 mm
2	B400C-16-TEMP-R-Prod. 1	161.0 HV	250 HV	0.78 mm	0.54 mm	12.47 mm
3	B400C-20-TEMP-R-Prod. 1	161.5 HV	250 HV	0.92 mm	0.67 mm	15.34 mm
4	B450C-16-TEMP-R-Prod. 1	186.0 HV	271 HV	1.51 mm	0.93 mm	10.11 mm
5	B450C-16-TEMP-R-Prod. 1	173.5 HV	266 HV	0.96 mm	0.83 mm	11.52 mm
6	B450C-16-TEMP-R-Prod. 1	155.0 HV	247 HV	1.01 mm	1.57 mm	10.75 mm
7	B450C-16-TEMP-R-Prod. 2	166.5 HV	257 HV	0.82 mm	0.78 mm	12.20 mm
8	B450C-20-TEMP-R-Prod. 2	167.0 HV	267 HV	1.25 mm	1.69 mm	13.50 mm
9	B450C-25-TEMP-R-Prod. 2	165.0 HV	266 HV	1.90 mm	2.10 mm	15.65 mm
10	B500B-16-TEMP-R-Prod. 1.2	177.0 HV	271 HV	1.16 mm	0.79 mm	11.26 mm
11	B500B-16-TEMP-R-Prod. 1.3	174.0 HV	266 HV	1.14 mm	1.20 mm	10.79 mm
12	B500B-16-TEMP-R-Prod. 1.1	182.0 HV	276 HV	1.47 mm	0.69 mm	10.60 mm
13	B500B-16-TEMP-R-Prod. 2	170.0 HV	267 HV	1.22 mm	1.30 mm	10.49 mm
14	B500B-20-TEMP-R-Prod. 2	172.5 HV	266 HV	1.58 mm	1.47 mm	13.12 mm
15	B500B-25-TEMP-R-Prod. 2	173.0 HV	271 HV	1.94 mm	1.87 mm	16.30 mm

**Table 3:** Measured bars properties (MA, CW and STR specimens).

Specimen (tag)		Hardness (HV)	FGS (μm)
16	B400C-16-MA-R-Prod.2	184	9.01
17	B400C-20-MA-R-Prod.2	178	8.64
18	B400C-25-MA-R-Prod.2	182	12.53
19	B500A-8-CW-R-Prod.2	196	12.1
20	B500A-8-CW-R-Prod.2	196	13.98
21	B500A-8-CW-I-Prod.1	201	11.08
22	B500A-12-CW-I-Prod.2	205	14.04
23	B500A-12-CW-I-Prod.2	202	14.04
24	B450C-8-STR-R-Prod.1	195	5.79
25	B500B-8-STR-R-Prod.1	208	7.84
26	B450C-12-STR-R-Prod.2	186	12.09
27	B450C-12-STR-R-Prod.2	199	8.04

#### 2.1.2 Monotonic tensile tests

Tensile tests were executed according to EN 15630-1:2010 [2] using a servo-hydraulic testing machine at University of Pisa laboratory. Force was measured using a load cell; for the evaluation of deformations, displacement sensors were directly positioned on the bar. Three tensile tests for each type of steel reinforcement were executed on specimens of adequate length (600 mm). Table 4 presents the averaged values of the achieved mechanical properties (yielding and tensile strength – *R*_e_, *R*_m_, elongation to maximum load and ultimate elongation – *A*_gt_, *A*_5_) and the corresponding standard deviations.

**Table 4:** Mechanical properties of tested rebars (monotonic tensile tests). Data refer to average value of three tests.

Steel grade/diameter/process/producer	*R*_m_ [MPa]	*σ*_Rm_	*R*_e_ [MPa]	*σ*_Re_	*R*_m_/*R*_e_	*σ*_Rm/Re_	*A* [%]	*σ*_A_	*A*_gt_ [%]	*σ*_Agt_
B400C-8-TEMP-R Prod.1	567.3	4.10	442.9	6.80	1.28	0.02	33.0	1.9	15.5	1.10
B400C-16-TEMP-R Prod.1	547.7	8.90	446.9	9.80	1.23	0.03	24.6	2.7	16.4	0.50
B400C-20-TEMP-R Prod.1	557.2	0.60	436.2	1.90	1.28	0.00	28.1	1.4	17.1	2.50
B400C-16-MA-R Prod.2	565.3	4.40	434.5	2.00	1.3	0.01	31.3	0.4	17.4	0.60
B400C-20-MA-R Prod.2	563.3	1.20	416.0	1.70	1.35	0.01	31.8	1.6	20.1	4.50
B400C-25-MA-R Prod.2	577.4	1.20	432.8	0.90	1.33	0.00	29.2	1.4	20.0	1.70
B450C-8-STR-R Prod.1	624.8	3.60	–	–	–	–	25.0	1.7	8.6	0.50
B450C-12-STR-R Prod.1	619.6	4.60	530.2	7.20	1.17	0.01	22.0	1.3	10.9	0.60
B450C-12-STR-R Prod.2	599.7	1.80	513.8	2.50	1.17	0.01	24.2	0.7	9.3	0.90
B450C-16-TEMP-R Prod.1(1)	640.5	32.2	537.3	27.1	1.19	0.01	23.9	1.3	8.9	1.10
B450C-16-TEMP-R Prod.1(2)	542.7	3.10	446.7	1.90	1.21	0.00	30.3	2.4	15.4	1.70
B450C-16-TEMP-R Prod.1(3)	615.4	2.50	517.8	5.60	1.19	0.01	25.4	0.2	13.8	1.50
B450C-16-TEMP-R Prod.2	601.1	4.80	479.3	14.6	1.25	0.03	28.8	0.5	17.5	1.62
B450C-20-TEMP-R Prod.2	591.4	9.70	492.9	9.00	1.20	0.03	27.3	1.5	14.1	0.20
B450C-25-TEMP-R Prod.2	629.8	6.40	505.0	2.90	1.25	0.02	24.5	1.0	14.2	0.60
B500A-8-CW-I Prod.1	581.3	6.10	582.3	9.90	1.00	0.03	17.3	3.0	3.2	0.70
B500A-8-CW-R Prod.2	546.8	6.70	526.4	1.60	1.04	0.01	19.5	1.0	6.0	0.60
B500A-12-CW-R Prod.2	589.0	4.80	567.7	9.40	1.04	0.01	20.5	2.3	7.5	0.60
B500B-8-TEMP-R Prod.2	671.5	36.3	584.7	26.9	1.15	0.03	15.3	1.8	8.3	2.10
B500B-8-STR-R Prod.1	619.3	10.5	565.6	8.90	1.10	0.01	21.3	1.1	4.5	0.60
B500B-12-TEMP-R Prod.1	627.0	5.20	538.4	7.60	1.16	0.015	18.3	1.2	10.5	0.80
B500B-16-TEMP-R Prod.1(1)	671.4	3.40	596.6	7.20	1.13	0.01	21.9	0.3	8.1	0.70
B500B-16-TEMP-R Prod.1(2)	668.3	14.3	572.0	12.0	1.17	0.00	24.0	0.8	11.3	0.70
B500B-16-TEMP-R Prod.1(3)	616.3	2.20	513.1	0.50	1.20	0.01	25.6	1.3	11.5	1.40
B500B-16-TEMP-R Prod.2	635.0	7.10	516.9	1.40	1.20	0.01	26.4	2.0	13.8	1.10
B500B-20-TEMP-R Prod.2	621.8	10.8	515.3	6.50	1.21	0.01	24.4	1.3	11.5	2.00
B500B-25-TEMP-R Prod.2	647.2	7.30	530.8	3.40	1.22	0.01	23.9	0.3	12.7	0.20

#### 2.1.3 Low-cycle fatigue tests

Low-Cycle Fatigue (LCF) tests are used to reproduce the effects of cyclic/seismic action: few tension/compression cycles with high imposed deformation. The assessment of the following parameters is needed to define an opportune testing protocol for LCF tests:

• Level of imposed deformation (*ε*).
• Testing frequency (*f*).
• Number of cycles to execute (*N*_cycles_).
• Length of the specimen (*L*_0_).

Analyzing data coming from actual scientific literature (Mander et al. [6]; Crespi [7]) and what provided by current standards for reinforcing steels (Portugal – LNEC E455–2008 [8]; Spain - UNE 36065 EX:2000 [9]), the following procedure was adopted:

• Two levels of imposed deformation: *ε*_1_=±2.5% and *ε*_2_=±4.0%.
• Testing frequency equal to 2.0 Hz. The value was reduced to 0.05 Hz for bar of large diameter after having evaluated the influence of strain rate on achieved data.
• Number of cycles to execute up to failure.
• Length of the specimen equal to stirrups’ spacing for new constructions: *L*_0H_=6*ϕ* and *L*_0L_=8*ϕ*.

LCF tests were executed in displacement control (Δ*l*) with a servo-hydraulic machine with load capacity equal to 250 kN. Deformations were directly measured from the machine, later depurating the values by the machine's deformability contribution according to what presented by Bray and Vicentini [10]. The level of elongation imposed to the bar, the free length and the testing frequency are summarized in Table 5. For each level of imposed deformation and specimen length two tests were executed.

**Table 5:** Testing parameters for LCF tests of different rebars' diameters.

*ϕ* [mm]	*f* [Hz]	Free length *L*_0_		Δε [%]	Δ*L* [mm]	Free length *L*_0_		Δε [%]	Δ*L* [mm]
8	2.0	6*ϕ*	48	±2.5	1.20	8*ϕ*	64	±2.5	1.60
				±4.0	1.92			±4.0	2.56
12	2.0	6*ϕ*	72	±2.5	1.80	8*ϕ*	96	±2.5	2.40
				±4.0	2.88			±4.0	3.84
16	2.0	6*ϕ*	96	±2.5	2.40	8*ϕ*	128	±2.5	3.20
				±4.0	3.84			±4.0	5.12
20	0.05	6*ϕ*	120	±2.5	3.00	8*ϕ*	160	±2.5	4.00
				±4.0	4.80			±4.0	6.40

Dissipated energy (W) and number of cycles up to failure (*N*_cycles_) were evaluated. The dissipated energy density per cycle was evaluated according to Apostolopoulos and Michalopoulos [11], as an approximation from the engineering stress–strain curves, according to Eq. (1).

(1) W=∫σdε

**Fig. 3:**
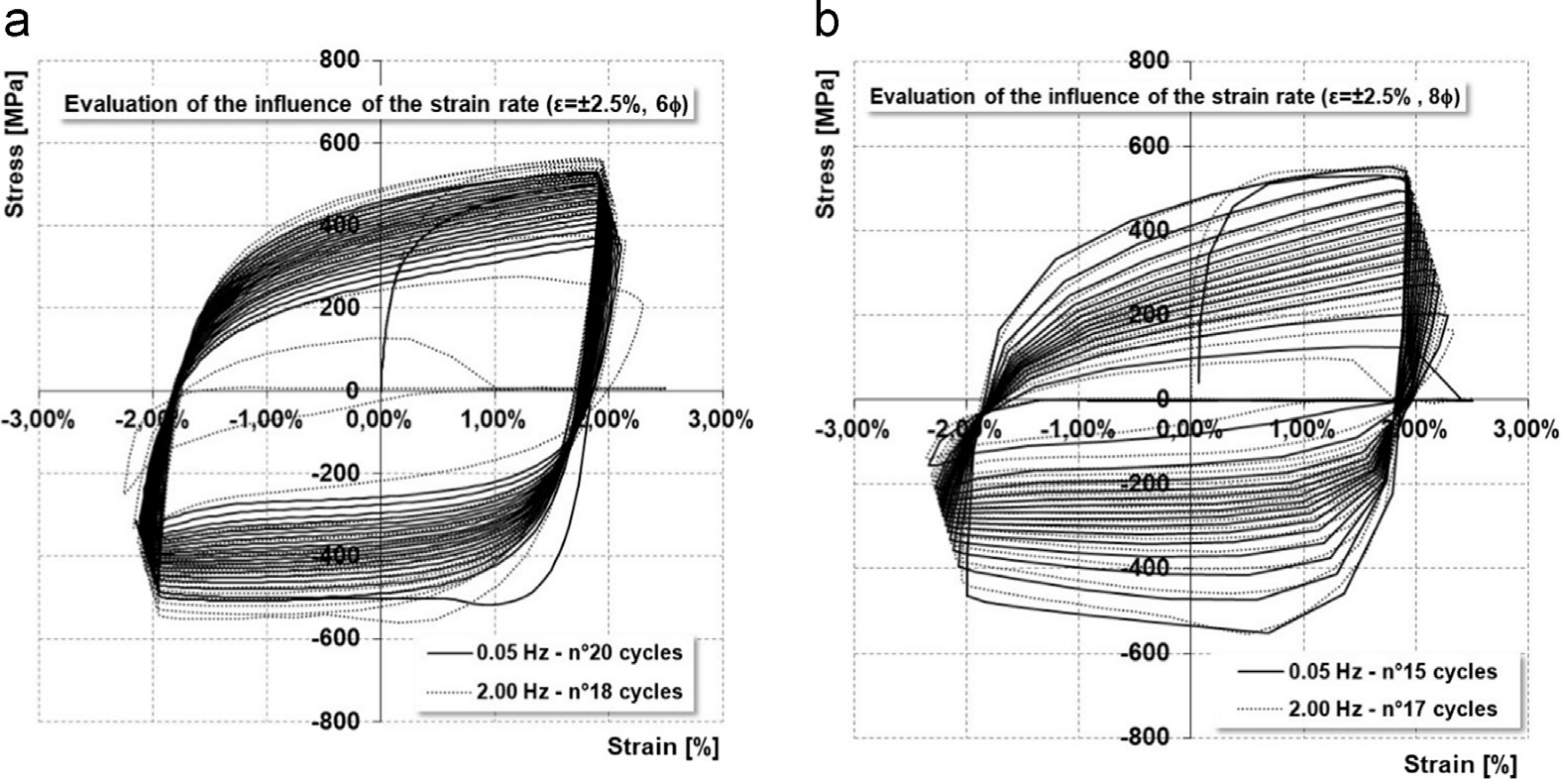
Stress–strain LCF curves for B450C-TEMP-16 (prod. 2) for ±2.5%, length 6*ϕ* (a) and 8*ϕ* (b) frequency 0.05 and 2.0 Hz.

Preliminary tests on B450C-16-TEMP-R bars allowed to assess the strain-rate influence on the cyclic performance, justifying the reduction of the testing frequency for large diameters. The difference in terms of total dissipated energy is presented in Table 6; percentage variations were evaluated excluding last cycles strongly suffering from damage and deterioration. A graphical representation is shown in Fig. 3. Data coming from LCF tests have been used to calibrate models for numerical simulations [12], [13].

**Table 6:** Experimental data coming from LCF tests for the assessment of strain-rate influence on B450C-16-TEMP-R.

Cycle no. [dimensionless]	*L*_0_=6*ϕ*			*L*_0_=8*ϕ*		
	Energy/cycle [MPa]		Difference[%]	Energy/cycle [MPa]		Difference[%]
	2.0 Hz	0.05 Hz		2.0 Hz	0.05 Hz	
1	31.67	32.96	3.91	31.58	33.74	6.39
2	31.54	33.02	4.49	31.67	29.82	6.18
3	30.9	32.43	4.71	28.29	27.78	1.83
4	29.44	31.76	7.29	25.68	25.21	1.86
5	29.33	31.1	5.67	23.68	23.25	1.84
6	28.35	30.51	7.07	22.1	21.7	1.87
7	27.84	29.92	6.93	20.79	20.38	2.01
8	27.28	29.36	7.11	19.63	19.16	2.41
9	26.22	28.81	8.99	18.54	17.99	3.05

**Fig. 4:**
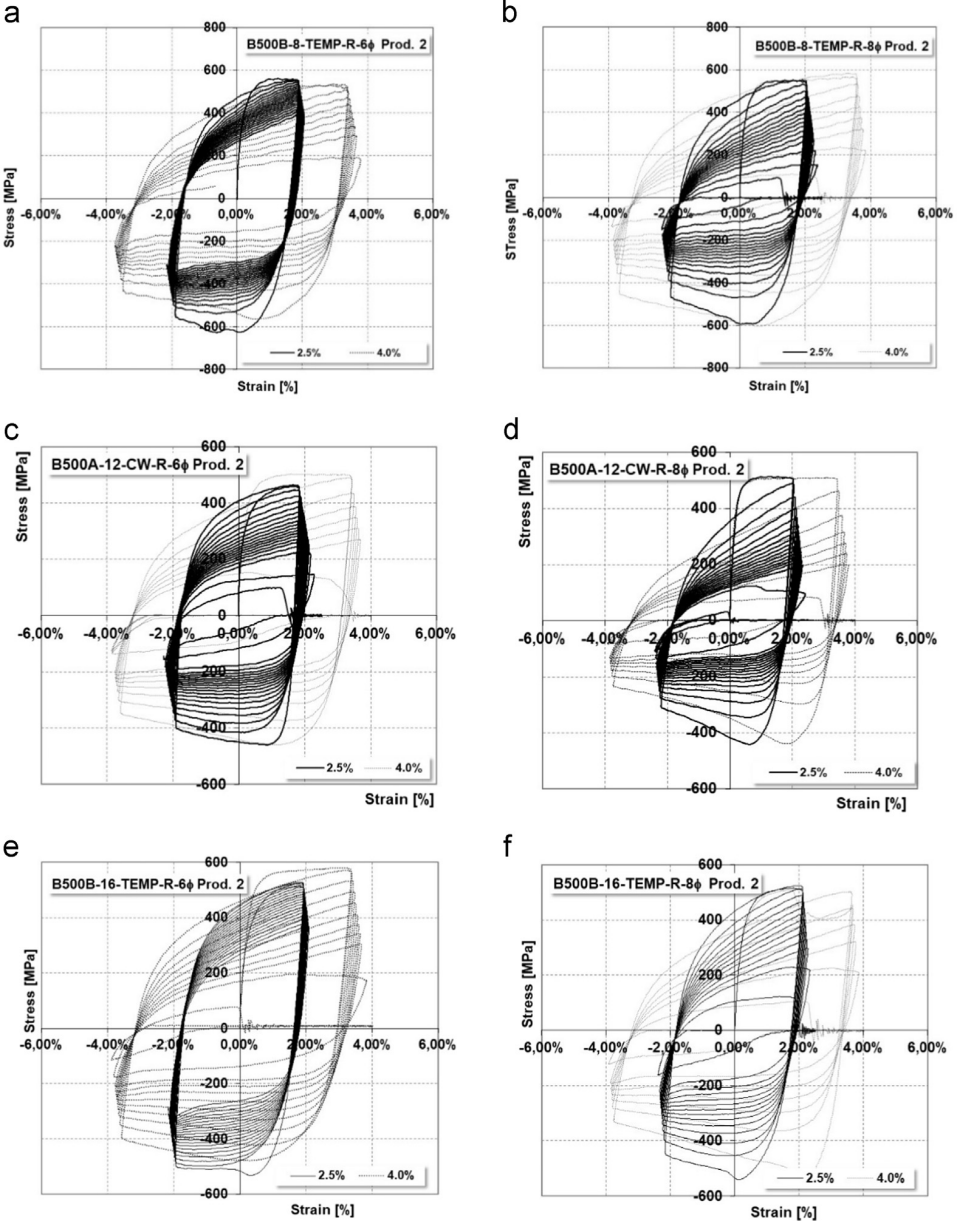
Example of stress–strain cyclic curves for different typologies of reinforcements.

Data coming from experimental LCF tests on specimens listed in Table 1 are summarized in Table 7, Table 8 respectively for *L*_0_ equal to 6*ϕ* and 8*ϕ*. Data are presented in terms of maximum and minimum tension/compression stresses, total dissipated energy and number of cycles to failure. Average values of the executed tests are presented, since data were perfectly aligned. Fig. 4 shows several stress–strain curves coming from LCF tests.

**Table 7:** LCF tests on bars for *L*_0_=6*ϕ*.

Steel type	*L*_0_	*f*[Hz]	Δ*ε*[%]	Max *σ*[MPa]	Min *σ*[MPa]	Energy[MPa]	*N*_cycles_[dimensionless]	Δ*ε*[%]	Max *σ*[MPa]	Min *σ*[MPa]	Energy[MPa]	*N*_cycles_[dimensionless]
B400C-8-TEMP-R-Prod.1	6*ϕ*	2	±2.5	472.1	−488.0	393.2	20	±4.0	499.5	−482.2	393.2	12
B450C-8-STR-R-Prod.1	6*ϕ*	2	±2.5	511.3	−490.6	456.1	20	±4.0	516.3	−456.1	428.7	11
B500A-8-CW-I-Prod.1	6*ϕ*	2	±2.5	522.5	−505.0	427.5	20	±4.0	531.7	−456.0	328.0	15
B500A-8-CW-R-Prod.2	6*ϕ*	2	±2.5	495.5	−627.8	498.3	20	±4.0	499.1	−434.3	322.6	14
B500B-8-STR-R-Prod.1	6*ϕ*	2	±2.5	562.8	−627.8	498.3	20	±4.0	537.3	−564.4	356.8	11
B500B-8-STR-R-Prod.1	6*ϕ*	2	±2.5	558.4	−487.6	458.7	20	±4.0	567.7	−520.4	376.9	12
B500A-12-CW-R-Prod.2	6*ϕ*	2	±2.5	464.8	−459.8	355.6	20	±4.0	502.8	−459.8	255.6	8
B450C-12-STR-R-Prod.2	6*ϕ*	2	±2.5	492.4	−452.1	446.2	20	±4.0	544.6	−388.5	341.6	14
B400C-16-TEMP-R-Prod. 1	6*ϕ*	2	±2.5	467.9	−452.2	385.8	18	±4.0	488.3	−437.4	276.4	8
B400C-16-MA-R-Prod. 2	6*ϕ*	2	±2.5	466.3	−465.9	429.6	20	±4.0	450.8	−466.0	418.7	12
B450C-16-TEMP-R-Prod.1.1	6*ϕ*	2	±2.5	616.7	−575.4	558.7	19	±4.0	631.2	−591.8	378.1	9
B450C-16-TEMP-R-Prod.1.3	6*ϕ*	2	±2.5	537.7	−557.8	532.2	19	±4.0	465.5	−515.4	551.0	14
B450C-16-TEMP-R-Prod.1.2	6*ϕ*	2	±2.5	483.4	−508.7	516.9	18	±4.0	550.7	−483.9	726.0	18
B450C-16-TEMP-R-Prod. 2	6*ϕ*	2	±2.5	562.5	−560.1	477.8	18	±4.0	552.4	−555.1	330.0	8
B500B-16-TEMP-R-Prod. 1.1	6*ϕ*	2	±2.5	565.6	−571.8	488.4	19	±4.0	583.6	−586.7	328.9	8
B500B-16-TEMP-R-Prod. 1.2	6*ϕ*	2	±2.5	577.7	−605.1	570.7	19	±4.0	583.0	−601.3	338.8	8
B500B-16-TEMP-R-Prod. 1.3	6*ϕ*	2	±2.5	530.3	−534.5	529.9	19	±4.0	572.6	−543.1	407.7	9
B500B-16-TEMP-R-Prod. 2	6*ϕ*	2	±2.5	529.5	−532.1	488.4	20	±4.0	580.3	−478.1	355.5	11
B400C-20-TEMP-R-Prod. 1	6*ϕ*	0.05	±2.5	411.3	−416.9	407.6	20	±4.0	458.1	−436.3	230.3	7
B400C-20-MA-R-Prod. 2	6*ϕ*	0.05	±2.5	430.4	−449.0	431.3	20	±4.0	495.1	−501.1	351.3	9
B450C-20-TEMP-R-Prod. 2	6*ϕ*	0.05	±2.5	497.7	−521.4	493.4	19	±4.0	521.5	−535.9	283.8	7
B500B-20-TEMP-R-Prod. 2	6*ϕ*	0.05	±2.5	570.5	−511.4	540.6	20	±4.0	597.5	−504.7	363.9	9

**Table 8:** LCF tests on bars for *L*_0_=8*ϕ*.

Steel type	*L*_0_	*f*[Hz]	Δε[%]	Max *σ*[MPa]	Min *σ*[MPa]	Energy[MPa]	*N*_cycles_[dimensionless]	Δ*ε*[%]	Max *σ*[MPa]	Min *σ*[MPa]	Energy[MPa]	*N*_cycles_[dimensionless]
B400C-8-TEMP-R-Prod.1	8*ϕ*	2	±2.5	461.5	−460.5	306.0	20	±4.0	487.4	−435.2	293.6	12
B450C-8-STR-R-Prod.1	8*ϕ*	2	±2.5	504.8	−415.4	339.4	20	±4.0	525.3	−410.7	332.0	16
B500A-8-CW-R-Prod.2	8*ϕ*	2	±2.5	512.9	−432.0	246.8	19	±4.0	514.3	−395.7	226.9	12
B500A-8-CW-I-Prod.1	8*ϕ*	2	±2.5	528.6	−460.0	273.8	19	±4.0	544.8	−471.1	237.5	10
B500B-8-STR-R-Prod.1	8*ϕ*	2	±2.5	553.4	−594.2	312.7	17	±4.0	584.8	−604.7	317.8	9
B500B-8-STR-R-Prod.1	8*ϕ*	2	±2.5	571.3	−454.3	334.2	20	±4.0	582.3	−458.7	277.4	10
B450C-12-STR-R-Prod.2	8*ϕ*	2	±2.5	495.3	−427.0	351.0	20	±4.0	506.3	−361.7	270.3	12
B500A-12-CW-R-Prod.2	8*ϕ*	2	±2.5	513.4	−441.7	250.4	17	±4.0	509.2	−439.8	187.2	8
B400C-16-TEMP-R Prod. 1	8*ϕ*	2	±2.5	461.3	−442.7	258.2	15	±4.0	463.0	−403.9	245.3	10
B400C-16-MA-R Prod. 2	8*ϕ*	2	±2.5	535.7	−418.3	377.6	17	±4.0	475.2	−445.5	211.1	8
B450C-16-TEMP-R Prod.1.1	8*ϕ*	2	±2.5	572.2	−607.3	261.5	13	±4.0	613.9	−540.8	471.5	11
B450C-16-TEMP-R Prod.1.3	8*ϕ*	2	±2.5	501.9	−551.0	292.0	15	±4.0	598.1	−491.9	380.0	9
B450C-16-TEMP-R Prod.1.2	8*ϕ*	2	±2.5	482.5	−508.4	353.7	18	±4.0	494.7	−477.1	230.3	9
B450C-16-TEMP-R Prod. 2	8*ϕ*	2	±2.5	531.5	−502.2	316.7	18	±4.0	510.5	−471.7	224.5	7
B500B-16-TEMP-R Prod. 1.1	8*ϕ*	2	±2.5	560.4	−566.4	293.6	15	±4.0	625.7	−510.0	360.7	10
B500B-16-TEMP-R Prod. 1.2	8*ϕ*	2	±2.5	564.8	−585.2	325.6	15	±4.0	587.7	−541.8	212.8	6
B500B-16-TEMP-R Prod. 1.3	8*ϕ*	2	±2.5	513.8	−502.5	268.3	13	±4.0	550.21	−524.3	246.6	8
B500B-16-TEMP-R Prod. 2	8*ϕ*	2	±2.5	524.1	−540.3	285.1	14	±4.0	506.9	−537.3	213.2	7
B400C-20-TEMP-R-Prod. 1	8*ϕ*	0.5	–	–	–	–	–	±4.0	368.0	−430.8	182.5	7
B400C-20-MA-R-Prod. 2	8*ϕ*	0.5	±2.5	466.2	−450.7	320.9	18	±4.0	446.8	−438.9	231.4	9
B450C-20-TEMP-R-Prod. 2	8*ϕ*	0.5	±2.5	509.3	−532.4	411.1	19	±4.0	493.8	−531.2	212.2	7
B500B-20-TEMP-R-Prod. 2	8*ϕ*	0.5	±2.5	545.5	−518.4	362.1	16	±4.0	509.5	−503.2	222.8	7

### 2.2 Experimental characterization of corroded steel reinforcing bars

Accelerated corrosion tests in salt-spray chamber were executed on a set of steel rebars reduced respect to the one presented in Table 1, as summarized in Table 9. On corroded samples monotonic tensile and low-cycle fatigue (LCF) tests were performed, comparing data achieved with reference (uncorroded) data.

**Table 9:** Reduced set of rebars subjected to accelerated corrosion tests. Indications about mechanical tests executed.

Steel grade	Ductility	Diameter	Process	Ribs	Producer	Tests performed
B400	C	16	TEMP	Ribbed (R)	Prod.1	Tensile+LCF
B400	C	16	MA	Ribbed (R)	Prod.2	Tensile+LCF
B400	C	25	MA	Ribbed (R)	Prod.2	Tensile
B450	C	12	STR	Ribbed (R)	Prod.1	Tensile+LCF
B450	C	16	TEMP	Ribbed (R)	Prod.1	Tensile+LCF
B450	C	25	TEMP	Ribbed (R)	Prod.2	Tensile
B500	A	12	CW	Ribbed (R)	Prod.2	Tensile+LCF
B500	B	12	STR	Ribbed (R)	Prod.1	Tensile
B500	B	16	TEMP	Ribbed (R)	Prod.1	Tensile+LCF
B500	B	25	TEMP	Ribbed (R)	Prod.2	Tensile

Salt-spray chamber test was selected as the most performing methodology to reproduce corrosion effects, due to time reasons and, besides, easiness of the preparation of the sample, following a codified standard (ISO 9227:2006 [14]). Two exposure periods were selected (45 and 90 days); tests were performed by three different Italian Laboratories in the following individuated as Laboratory 1, 2 and 3. The adopted protocol can be schematized into the following steps.

**Fig. 5:**
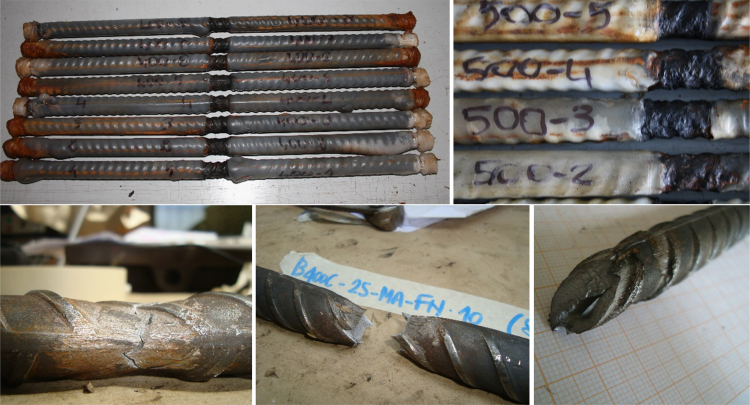
Specimens after corrosion in salt-spray chamber (Lab. 1) and after execution of tensile tests.

• *Step 1: Preparation of the testing apparatus*. The chamber, piping and solution tank shall be perfectly cleaned from previous experiments reaching Ph values between 5.5 and 6.2. Before placing the specimens, at least 50 l of solution shall circulate for about 6 h under the pre-determined wet/dry cycle to stabilize the pH of the chamber. These prescriptions are aligned to ISO 9227:2006 standard.
• *Step 2: Preparation of the specimens*. Specimens shall have a length between 500 and 600 mm to execute tensile tests after corrosion determining the stress–strain diagram. In the middle section of the specimen a high temperature aluminum (non-adhesive) tape shall be placed; the tape has a width equal to about 20 mm or, at least, the distance between two following ribs: this length is the ‘unprotected’ part of the specimen, exposed to corrosion. The other portion of the bar is otherwise protected by a natural wax covering
• *Step 3: Tests' execution.* The specimens shall be placed at an angle of 45–60° to the supports, rotating them by 90° at least three times a day to prevent salts' generation, according to ISO 9227:2006 [14], for the full duration of the tests. At least 8 wet/dry cycles shall be programmed per 24 h (90 min dry and 90 min wet). The Ph shall be monitored for the whole test's duration (i.e. 45 and 90 days).
• *Step 4: Measurement of the corrosion damage before experimental tests.* After the tests, specimens shall be rubbed with a fine steel brush and cleaned with tap water, keeping attention to prevent heat generation. Mass loss shall be measured on corroded specimens: this is the most relevant corrosion indicator.
• *Step 8: Execution of Mechanical tests on corroded rebars.* Experimental tensile and Low-Cycle Fatigue tests shall be performed according to what already presented on corroded specimens, also measuring the notch depth, crack depth and width and, mainly, cross-section reduction (necking) after monotonic tests (Fig. 5).

#### 2.2.1 Monotonic tensile tests on corroded specimens

Data coming from tensile tests on corroded specimens are presented in terms of mechanical properties (*R*_e_, *R*_m_, *A*_gt_ and *A*) and mass loss (ML). Mass loss was evaluated as ratio the between the mass variation before and after corrosion (Δ*M*=*M*_i_−*M*_f_) and the initial mass of the effective exposed length (*M*_uncorr_), according to Eq. (2). This kind of measure is needed since *L*_corr_ can vary due to practical operations during the preparation phase.

(2) $$\mathrm{ML}=\frac{∆M}{M_{\mathrm{uncorr}}}=\frac{M_{i}-M_{f}}{M_{\mathrm{uncorr}}}$$

Necking (*Z*) of the cross-section area was evaluated after tensile tests. The percentage variation of the necking (Δ*Z*), for each corroded specimen, was evaluated according to Eq. (3), being *Z*_*uncorr*_ and *Z*_*corr*_ respectively the necking of specimens before and after corrosion. For reference specimens a mean value was assumed, considering the presence of ribs.

(3) $$\Delta Z=\frac{Z_{corr}-Z_{uncorr}}{Z_{uncorr}}$$

**Fig. 6:**
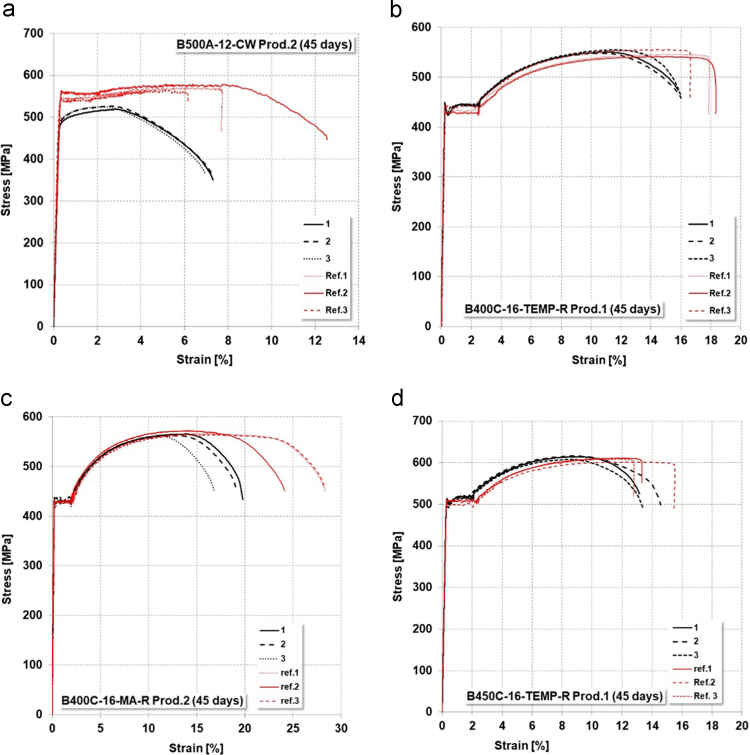

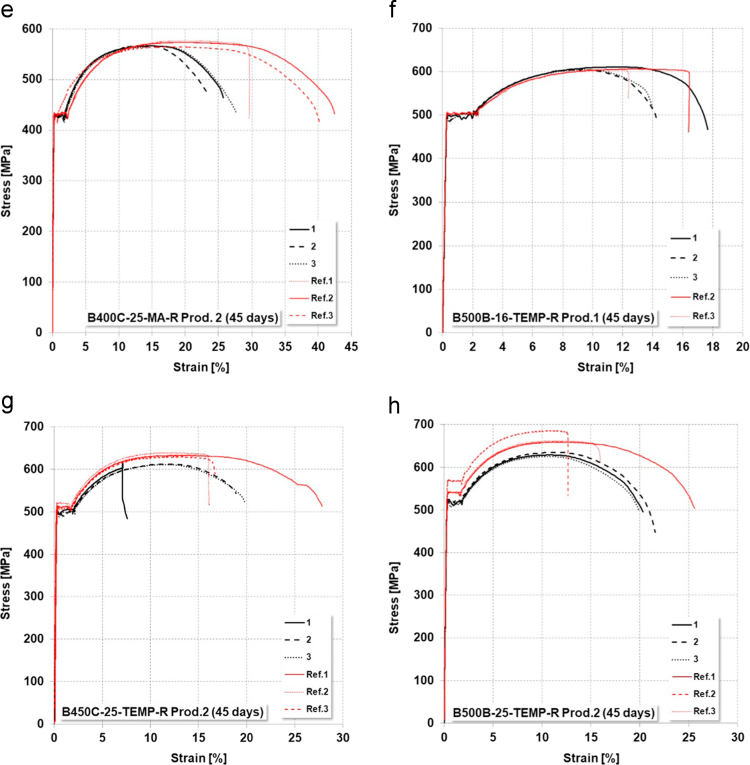
Stress–strain curves of corroded specimens in comparison to reference rebars (45 days salt-spray): (a) B500A-12-CW- Prod.1; (b) B400C-16-TEMP-Prod.1; (c) B400C-16-MA-Prod.2; (d) B450C-16-TEMP-Prod.1; (e) B400C-25-MA-Prod. 2; (f) B500B-16-TEMP- Prod.1; (g) B450C-25-TEMP-Prod.2; (h) B500B-25-TEMP-Prod.2.

**Fig. 7:**
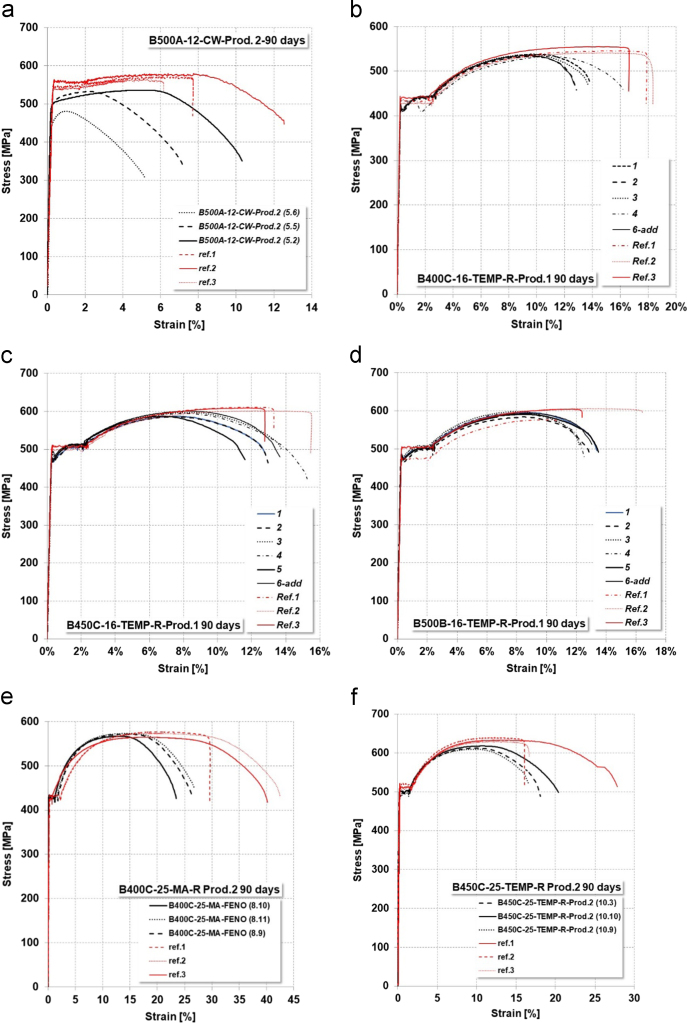
Stress–strain curves of corroded specimens in comparison to reference rebars (90 days salt-spray): (a) B500A-12-CW-Prod.2; (b) B400C-16-TEMP-Prod.1; (c) B450C-16-TEMP-Prod.1; (d) B500B-16-TEMP-R-Prod.1; (e) B400C-25-MA-R-Prod.2; (f) B450C-25-TEMP-R-Prod. 2.

Data achieved from tensile tests on corroded steel rebars are presented in Table 10, Table 11 for respectively 45 and 90 days of exposure. Tests were performed in three different laboratories (ILVA S.p.A – Lab.1, Bavaro laboratory – Lab. 2, Omeco laboratory – Lab. 3). Fig. 6, Fig. 7 presents several stress–strain curves achieved from tensile tests on corroded specimens, compared to reference ones (uncorroded condition) ).

**Table 10:** Tensile test on corroded rebars after 45 days of salt-spray chamber.

45 days of exposure	*L*_corr_ [mm]	Δ*Μ* [g]	ML [%]	*R*_e_ [MPa]	*R*_m_ [MPa]	*R*_m_/*R*_e_ [dimensionless]	*A*_gt_ [%]	*A*_5_ [%]	Δ*Ζ* [%]	Lab
B500A-12-CW-I-Prod.2–1	21.7	3.26	17.0	489.5	512.3	1.05	1.3	11.8	−19	1
B500A-12-CW-I-Prod.2–2	23.3	2.91	14.0	495.0	518.6	1.05	0.9	10.7	−12	1
B500A-12-CW-I-Prod.2–3	21.5	4.23	22.0	498.6	517.8	1.04	0.8	10.8	−24	1
B400C-16-TEMP-R-Prod.1–1	31.0	4.79	10.0	444.5	550.2	1.24	8.4	19.6	−8	1
B400C-16-TEMP-R-Prod.1–2	30.5	6.34	13.0	449.2	548.2	1.22	7.5	17.5	−8	1
B400C-16-TEMP-R-Prod.1–3	31.7	7.47	15.0	436.5	554.6	1.27	9	17.6	−10	1
B400C-16-MA-R-Prod.2–1	31.5	5.98	12.0	427.2	562.1	1.32	10.6	21.6	−19	1
B400C-16-MA-R-Prod.2–2	29.5	5.05	11.0	437.5	562.0	1.28	9.8	21	−9	1
B400C-16-MA-R-Prod.2–3	31.2	11.19	22.0	424.0	560.0	1.32	10.3	20.9	−30	1
B450C-16-TEMP-R-Prod.1–1	30.5	3.87	8.0	509.2	614.3	1.21	6.9	16.4	−32	1
B450C-16-TEMP-R-Prod.1–2	29.5	3.54	7.0	511.2	615.9	1.2	6.2	16.9	−18	1
B450C-16-TEMP-R-Prod.1–3	28.8	5.18	11.0	504.3	607.9	1.21	5.7	16.4	−24	1
B500B-16-TEMP-R-Prod.1–1	31.5	10.57	21.0	500.0	610.3	1.22	9.1	19.4	−18	1
B500B-16-TEMP-R-Prod.1–2	31.2	9.62	19.0	490.9	604.3	1.23	6.3	17.8	−22	1
B500B-16-TEMP-R-Prod.1–3	23.2	9.55	26.0	492.0	604.2	1.23	7.5	16.5	−23	1
B400C-25-MA-R-Prod.2–1	25.9	0.66	1.0	427.5	575.7	1.35	11.6	20	−20	1
B400C-25-MA-R-Prod.2–2	22.15	0.75	1.0	425.8	576.2	1.35	12.7	14	−20	1
B400C-25-MA-R-Prod.2–3	21.85	0.62	1.0	424.0	576.0	1.36	13.3	15.7	−16	1
B450C-25-TEMP-R-Prod.2–1	22.0	0.3	0.0	500.3	622.1	1.24	9.1	19.8	−15	1
B450C-25-TEMP-R-Prod.2–2	25.5	0.7	1.0	495.0	618.1	1.25	8.3	19.2	−8	1
B450C-25-TEMP-R-Prod.2–3	22.9	0.7	1.0	497.4	617.2	1.24	8.5	18.2	−7	1
B500B-25-TEMP-R-Prod.2–1	26.4	1.72	2.0	518.4	637.1	1.23	8.5	19.2	−2	1
B500B-25-TEMP-R-Prod.2–2	23.0	1.67	2.0	524.3	643.2	1.23	9.3	18.2	−8	1
B500B-25-TEMP-R-Prod.2–3	24.2	11.99	2.0	513.7	633.6	1.23	8.2	18.1	−5	1

**Table 11:** Tensile test on corroded rebars after 90 days of salt-spray chamber.

90 days of exposure	*L*_corr_ [mm]	Δ*Μ* [g]	ML [%]	*R*_e_ [MPa]	*R*_m_ [MPa]	*R*_m_/*R*_e_ [dimensionless]	*A*_gt_ [%]	*A*_5_ [%]	Δ*Ζ* [%]	Lab	
B500A-12-CW-I-Prod.2 5.6	24.9	2.7	12.6	461.0	480.0	1.04	0.9	13.3	−27		2
B500A-12-CW-I-Prod.2 5.5	21.0	0.8	4.5	508.0	532.6	1.05	2.4	14.7	−17		2
B500A-12-CW-I-Prod.2 5.2	182.5	8.4	5.3	505.0	535.0	1.06	5.1	14.2	−24		2
B400C-16-TEMP-R-Prod.1–1	30.0	6.3	13.5	398.4	525.3	1.32	7.1	17.1	−45		1
B400C-16-TEMP-R-Prod.1–2	28.4	8.3	18.9	401.4	520.6	1.30	5.8	14.8	35		1
B400C-16-TEMP-R-Prod.1–3	30.0	5.6	12.2	404.9	524.8	1.30	6.4	15.1	−32		1
B400C-16-TEMP-R-Prod.1–4	24.9	6.1	15.9	417.3	518.5	1.24	7.5	19.4	−34		1
B400C-16-TEMP-R-Prod.1–5	25.3	6.2	16.0	410.8	–	–	7.6	16.8	−45		1
B400C-16-TEMP-R-Prod.1–6	25.1	8.3	21.6	414.6	522.6	1.26	8.0	15.4	−14		1
B450C-16-TEMP-R-Prod.1–1	20.9	4.9	14.6	481.4	599.5	1.25	4.3	15.4	−17		1
B450C-16-TEMP-R-Prod.1–2	26.4	2.6	6.1	484.4	598.0	1.23	4.4	15.6	−13		1
B450C-16-TEMP-R-Prod.1–3	27.2	3.8	8.7	499.8	610.5	1.22	5.1	16.6	−25		1
B450C-16-TEMP-R-Prod.1–4	28.9	3.2	6.9	497.4	607.9	1.22	5.7	17.8	2%		1
B450C-16-TEMP-R-Prod.1–5	24.2	3.3	8.3	480.9	600.0	1.25	4.1	14.1	−15		1
B450C-16-TEMP-R-Prod.1–6	24.5	6.8	17.3	502.8	613.8	1.22	5.5	16.3	−19		1
B500B-16-TEMP-R-Prod.1–1	28.6	11.2	24.3	492.4	607.9	1.23	5.7	14.8	−6		1
B500B-16-TEMP-R-Prod.1–2	30.5	8.3	17.0	476.5	596.4	1.25	4.6	15.5	−15		1
B500B-16-TEMP-R-Prod.1–3	20.0	14.5	44.9	481.9	610.5	1.27	5.0	14.9	−22		1
B500B-16-TEMP-R-Prod.1–4	24.5	6.7	16.9	485.4	606.3	1.25	5.1	15.4	−21		1
B500B-16-TEMP-R-Prod.1–5	26.4	11.8	27.8	491.4	603.2	1.23	5.0	15.6	−6		1
B500B-16-TEMP-R-Prod.1–6	24.2	6.8	17.5	490.3	605.6	1.24	5.5	16.4	−14		1
B400C-25-MA-R-Prod.2 8.10	22.8	4.5	5.1	442.7	569.5	1.29	12.9	23.6	−13		2
B400C-25-MA-R-Prod.2 8.11	22.0	8.5	10.0	437.7	563.4	1.29	15.0	27.4	−6		2
B400C-25-MA-R-Prod.2 8.9	17.1	6.5	9.8	438.7	573.5	1.31	16.1	26.5	−15		2
B450C-25-TEMP-R-Prod.2 10.3	21.6	1.0	1.2	502.4	623.7	1.24	9.6	18.9	−10		2
B450C-25-TEMP-R-Prod.2 10.9	20.4	1.5	1.9	515.5	630.8	1.22	10.0	17.8	2		2
B450C-25-TEMP-R-Prod.2 10.10	21.6	7.0	8.4	515.5	628.8	1.22	8.5	19.3	−9		2
B500B-25-TEMP-R-Prod.2 6.9	21.7	3.0	3.6	533.1	640.1	1.20	8.8	18.6	−4		2
B500B-25-TEMP-R-Prod.2 6.1	23.2	18.5	20.6	537.1	646.2	1.20	8.1	18.6	−13		2
B500B-25-TEMP-R-Prod.2 6.8	22.8	2.5	2.8	535.1	640.1	1.20	8.7	19.9	−5		2

**Table 12:** LCF tests for length of the specimen equal to 6*ϕ* and imposed deformation ±2.5%.

90 days of exposure		*L*_0_ [mm]	Δ*ε* [%]	*f* [Hz]	ML [%]	Max *σ* [MPa]	Min *σ* [MPa]	Energy [MPa]	*N*_cycles_	Lab
B450C-12-STR-R-Prod.1	9.5	6*ϕ*	±2.5	0.5	8.2	555.0	−525.0	471	18	3
B450C-12-STR-R-Prod.1	9.1	6*ϕ*	±2.5	0.5	5.8	551.0	−536.0	591	20	3
B450C-12-STR-R-Prod.1	9.7	6*ϕ*	±2.5	0.5	28.9	561.0	−529.0	362	16	3
B400C-16-MA-R-Prod.2	4.8	6*ϕ*	±2.5	0.5	8.0	523.0	−519.0	449	17	2
B400C-16-MA-R-Prod.2	4.9	6*ϕ*	±2.5	0.5	6.7	519.0	−507.0	350	14	2
B400C-16-MA-R-Prod.2	4.11	6*ϕ*	±2.5	0.5	9.9	498.0	−485.0	305	13	2
B400C-16-TEMP-R-Prod.1	3.2	6*ϕ*	±2.5	0.5	2.5	482.0	−485.0	468	19	3
B400C-16-TEMP-R-Prod.1	3.4	6*ϕ*	±2.5	0.5	4.3	481.0	−475.0	424	17	3
B450C-16-TEMP-R-Prod.1	2.4	6*ϕ*	±2.5	0.5	7.7	512.5	−524.7	371	14	2
B450C-16-TEMP-R-Prod.1	2.6	6*ϕ*	±2.5	0.5	9.4	512.4	−528.2	371	15	2
B450C-16-TEMP-R-Prod.1	2.7	6*ϕ*	±2.5	0.5	7.3	509.7	−518.8	377	15	2
B500B-16-TEMP-R-Prod.1	1.2	6*ϕ*	±2.5	1	6.6	539.0	−565.0	537	21	2
B500B-16-TEMP-R-Prod.1	1.4	6*ϕ*	±2.5	1	7.9	536.0	−545.0	-553	19	2
B500B-16-TEMP-R-Prod.1	1.8	6*ϕ*	±2.5	1	7.2	536.0	−564.0	486	19	2

**Table 13:** LCF tests for length of the specimen equal to 6*ϕ* and imposed deformation ±4.0%.

90 days of exposure		*L*_0_ [mm]	Δ*ε* [%]	*f* [Hz]	ML [%]	Max *σ* [MPa]	Min *σ* [MPa]	Energy [MPa]	*N*_cycles_	Lab
B450C-12-STR-R-Prod.1	9.3	6*ϕ*	±4.0	0.5	8.9	589.0	−531.0	288	7	3
B450C-12-STR-R-Prod.1	9.14	6*ϕ*	±4.0	0.5	4.7	573.0	−522.0	346	8	3
B450C-12-STR-R-Prod.1	9.18	6*ϕ*	±4.0	0.5	3.3	587.0	−539.0	315	7	3
B400C-16-MA-R-Prod.2	4.12	6*ϕ*	±4.0	0.5	11.5	533.0	−514.0	314	8	2
B400C-16-MA-R-Prod.2	4.15	6*ϕ*	±4.0	0.5	8.5	537.0	−523.0	289	7	2
B400C-16-TEMP-R-Prod.1	3.6	6*ϕ*	±4.0	0.5	3.2	506.0	−482.0	335	8	3
B400C-16-TEMP-R-Prod.1	3.7	6*ϕ*	±4.0	0.5	2.3	509.0	−484.0	322	8	3
B450C-16-TEMP-R-Prod.1	2.1	6*ϕ*	±4.0	0.5	7.7	533.7	−518.7	307	7	2
B450C-16-TEMP-R-Prod.1	2.2	6*ϕ*	±4.0	0.5	9.5	516.8	−509.1	291	7	2
B450C-16-TEMP-R-Prod.1	2.3	6*ϕ*	±4.0	0.5	7.9	531.2	−521.1	307	7	2
B500B-16 -TEMP-R-Prod.1	1.10	6*ϕ*	±4.0	1	7.6	555.0	−578.0	295	7	2
B500B-16 -TEMP-R-Prod.1	1.12	6*ϕ*	±4.0	1	5.3	558.0	−551.0	440	12	2

**Table 14:** LCF tests for length of the specimen equal to 8*ϕ* and imposed deformation ±2.5%.

90 days of exposure		*L*_0_ [mm]	Δ*ε* [%]	*f* [Hz]	ML [%]	Max *σ* [MPa]	Min *σ* [MPa]	Energy [MPa]	*N*_cycles_	Lab
B450C-12-STR-R-Prod.1	9.12	8*ϕ*	±2.5	0.5	3.3	560.0	−488.0	265	12	3
B450C-12-STR-R-Prod.1	9.13	8*ϕ*	±2.5	0.5	7.3	556.0	−477.0	275	13	3
B450C-12-STR-R-Prod.1	9.17	8*ϕ*	±2.5	0.5	5.6	562.0	−483.0	514	12	3
B400C-16-MA-R-Prod.2	4.3	8*ϕ*	±2.5	0.5	7.1	500.0	−515.0	230	11	2
B400C-16-MA-R-Prod.2	4.4	8*ϕ*	±2.5	0.5	8.0	496.0	−506.0	247	12	2
B400C-16-MA-R-Prod.2	4.7	8*ϕ*	±2.5	0.5	7.5	492.0	−491.0	178	9	2
B400C-16-TEMP-Prod.1	3.12	8*ϕ*	±2.5	0.5	0.4	452.0	−467.0	277	15	3
B450C-16-TEMP-Prod.1	2.5	8*ϕ*	±2.5	0.5	8.2	498.1	−511.1	214	10	2
B450C-16-TEMP-Prod.1	2.10	8*ϕ*	±2.5	0.5	9.4	508.0	−484.7	215	11	2
B450C-16-TEMP-Prod.1	2.11	8*ϕ*	±2.5	0.5	9.2	513.4	−528.2	273	13	2
B450C-16-TEMP-Prod.1	2.12	8*ϕ*	±2.5	0.5	6.9	496.4	−507.2	290	16	2
B500B-16-TEMP-Prod.1	1.1	8*ϕ*	±2.5	1	7.1	540.0	−570.0	271	13	2
B500B-16-TEMP-Prod.1	1.6	8*ϕ*	±2.5	1	7.3	533.0	−553.0	223	8	2
B500B-16-TEMP-Prod.1	1.9	8*ϕ*	±2.5	1	5.9	519.0	−518.0	289	14	2

**Table 15:** LCF tests for length of the specimen equal to 8*ϕ* and imposed deformation ±4.0%.

90 days of exposure		*L*_0_ [mm]	Δ*ε* [%]	*f* [Hz]	ML [%]	Max *σ* [MPa]	Min *σ* [MPa]	Energy [MPa]	*N*_cycles_	Lab
B450C-12-STR-R-Prod.1	9.8	8*ϕ*	±4.0	0.5	4.1	574.0	−470.0	258	8	3
B450C-12-STR-R-Prod.1	9.9	8*ϕ*	±4.0	0.5	5.6	585.0	−471.0	207	7	3
B450C-12-STR-R-Prod.1	9.4	8*ϕ*	±4.0	0.5	8.6	591.0	−487.0	235	7	3
B400C-16-MA-R-Prod.2	4.1	8*ϕ*	±4.0	0.5	5.5	528.0	−511.0	216	6	2
B400C-16-MA-R-Prod.2	4.6	8*ϕ*	±4.0	0.5	6.4	527.0	−496.0	247	8	2
B400C-16-MA-R-Prod.2	4.10	8*ϕ*	±4.0	0.5	10.1	523.0	−507.0	197	6	2
B400C-16-TEMP-R-Prod.1	3.3	8*ϕ*	±4.0	0.5	6.2	456.0	−476.0	171	6	3
B400C-16-TEMP-R-Prod.1	3.5	8*ϕ*	±4.0	0.5	2.6	455.0	−486.0	219	8	3
B400C-16-TEMP-R-Prod.1	3.10	8*ϕ*	±4.0	0.5	4.7	480.0	−489.0	217	7	3
B450C-16-TEMP-R-Prod.1	2.8	8*ϕ*	±4.0	0.5	6.9	511.5	−479.4	207	6	2
B450C-16-TEMP-R-Prod.1	2.9	8*ϕ*	±4.0	0.5	6.5	503.9	−496.0	173	5	2
B500B-16-TEMP-R-Prod.1	1.11	8*ϕ*	±4.0	1.0	7.2	505.0	−529.0	203	7	2
B500B-16-TEMP-R-Prod.1	1.13	8*ϕ*	±2.5	1.0	6.1	536.0	−545.0	319	17	2

#### 2.2.2 Low-Cycle Fatigue (LCF) tests on corroded specimens

**Fig. 8:**
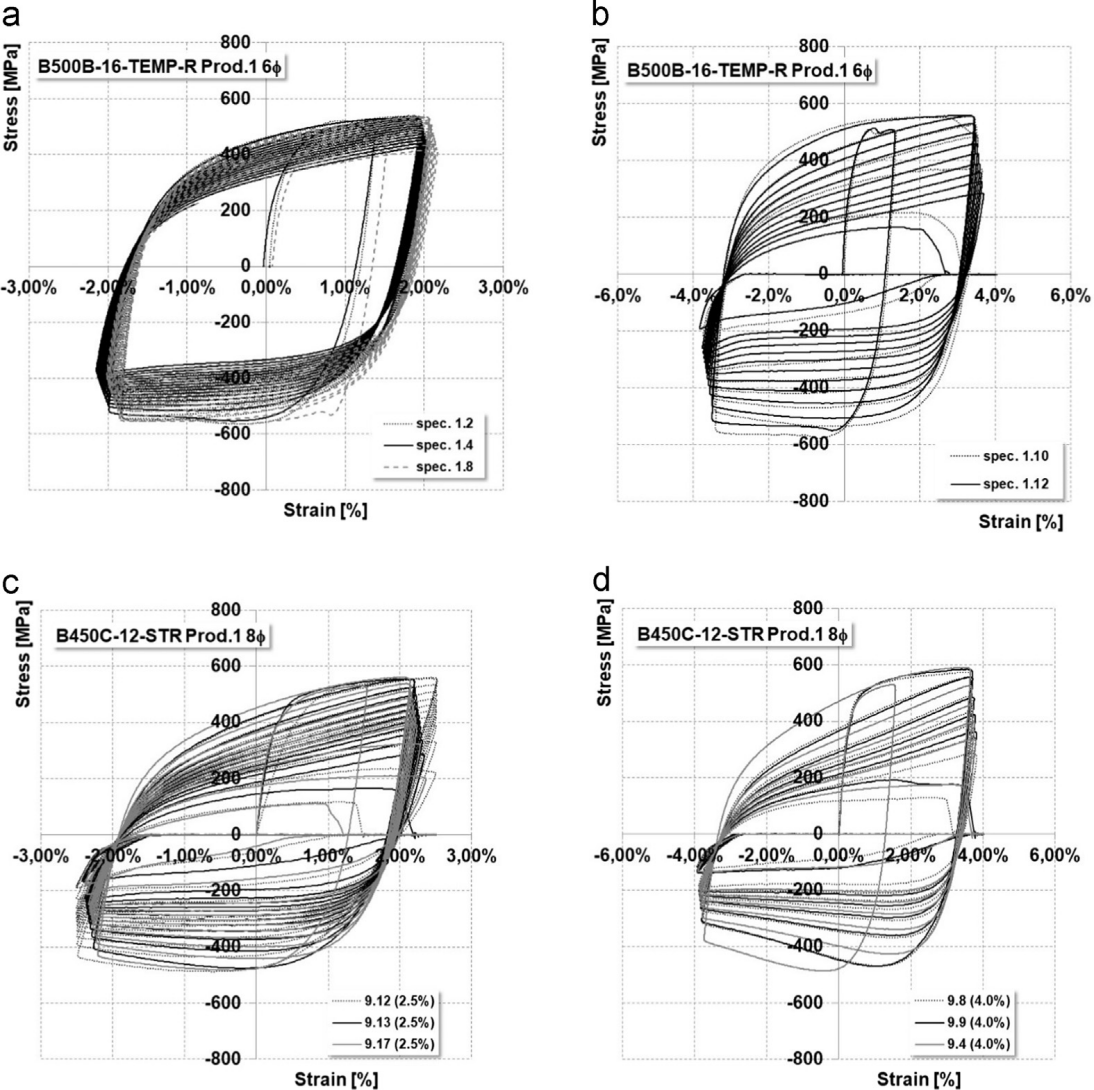
Example of stress–strain cyclic curves on corroded specimens.

Low-Cycle Fatigue (LCF) tests were executed on several corroded bars; the protocol already presented for uncorroded rebars was followed. Achieved data are presented in terms of ML, maximum and minimum effective deformation and stress, total dissipated energy and number of cycles up to failure (Fig. 8, Table 12, Table 13, Table 14, Table 15).
